# Transudative and Exudative Pleural Effusion in Chronic Kidney Disease Patients: A Prospective Single-Center Study

**DOI:** 10.7759/cureus.18649

**Published:** 2021-10-10

**Authors:** Asfia Jabbar, Ruqaya Qureshi, Kiran Nasir, Murtaza Dhrolia, Aasim Ahmad

**Affiliations:** 1 Nephrology, The Kidney Centre Post Graduate Training Institute, Karachi, PAK

**Keywords:** exudative pleural effusion, transudative pleural effusion, hemodialysis, pleural effusion, chronic kidney disease

## Abstract

Objective

The aim of the study is to assess the incidence of pleural effusion and to assess its etiology in admitted chronic kidney disease patients who were admitted secondary to various causes, i.e., fluid overload, sepsis, etc.

Material and methods

A prospective cross-sectional observational study was conducted at the Department of Nephrology, The Kidney Centre Postgraduate Training Institute, Karachi. A total of 789 patients were admitted between August 2020-February 2021. This study comprised 280 adult chronic kidney disease (chronic kidney disease and end-stage renal disease patients who were on dialysis) patients having pleural effusion (either unilateral or bilateral) secondary to various causes.

Results

Among 280 patients, the mean age was 55 years with 158 (56.4%) males and 122 (43.6%) females, diabetes (76%) was present in most of the patients along with hypertension (86.1%), and most of the patients were of stage IV and V. Transudative pleural effusion was present in 212 (75.7%) patients secondary to fluid overload and heart failure was the commonest cause while 68 (24.3%) patients had exudative pleural effusion with tuberculosis being the commonest etiology, 44 (15.7%) patients needed intervention while 236 (84.3%) were treated medically. The data was entered and analyzed on SPSS version 21 (IBM Corp, Armonk, USA). The cleaning and coding of data were done before analysis. Continuous variables were expressed in mean ± standard deviation, while the frequencies with percentages were obtained for categorical variables. The Chi-square test was applied to see the association between variables. A p-value of ≤ 0.05 was considered significant.

Conclusion

Clarification of the cause of pleural effusion is essential. Early diagnosis and prompt treatment like thoracocentesis or in the case of patients on hemodialysis, adequate dialysis may be necessary.

## Introduction

Chronic kidney disease (CKD) is recognized as a major health problem. Globally, the load of CKD is swiftly increasing, its estimated prevalence is about 13.4%, while in Pakistan more than 17 million people are suffering from kidney diseases [1]. The current international consensus definition of CKD [2] states that CKD is defined as abnormalities of kidney structure or function, present for > three  months, with implications for health. With time, patients with CKD develop various systemic complications and respiratory complications are of utmost importance and clinically significant. Pleural effusion (PE) and pulmonary edema are common clinical presentations in CKD patients [3], which occur mostly due to fluid overload and increased capillary permeability of visceral and parietal pleura.

PE is an excessive accumulation of fluid in the pleural spaces from a lack of balance between pleural fluid formation and evacuation. In patients with renal involvement, it is a common diagnostic dilemma as it may arise because of CKD itself. To treat PE, it’s important to know its etiology. PE can be unilateral or bilateral and can vary from mild to massive.

PE can be transudative or exudative and this can be diagnosed according to the Lights criteria [4]. The common causes of transudative pleural effusion are fluid overload, heart failure and nephrotic syndrome, while the causes for exudative pleural effusion are pneumonia, tuberculosis (TB), pulmonary embolisms or diseases causing pleuro-renal syndromes, like systemic lupus erythematosus. Another important cause is uraemic pleurisy (exudative PE) which is a diagnosis of exclusion, that persists or recurs despite aggressive hemodialysis (HD) [5].

Most studies looking into the incidence of PE in patients with CKD are retrospective studies and on long-term dialysis patients [6-8]. In the present study, we prospectively studied the occurrence, causes, clinical features and management issues of admitted CKD patients of all stages who had PE (stages II-V including dialysis population).

## Materials and methods

The study is a prospective cross-sectional observational analysis conducted at The Kidney Centre Post Graduate Training Institute, Karachi, Pakistan (TKC-PGTI) after getting approval from the hospital Ethical Review Committee (ERC Reference No. 94-NEPH-062020).

Inclusion criteria were all adult CKD (of all stages with or without dialysis) patients having PE (either unilateral or bilateral) who got admitted with various causes between August 2020 and February 2021. Patients who had acute kidney injury were excluded from the study. A total of 789 CKD patients were admitted to our institute for different reasons. We collected data from all admitted CKD patients on HD or not on HD with PE. Patients who met the inclusions criteria were included in the study. Patients’ demographic information and previous history were recorded using a pre-designed case report form. There was no direct interaction with patients in the study. Detailed demographic and clinical parameters including age, sex, smoking history, clinical symptoms with duration and clinical signs and other systemic examination for the comorbid illness were evaluated in all patients. Comorbid conditions were defined as the presence of coexisting cardiac failure, ischemic heart disease, chronic lung disease (COPD), chronic liver disease, malignancies and diabetes mellitus.

We gathered all the following data: clinical findings including shortness of breath (SOB), chest pain, fever, cough, sputum and laboratory data including complete blood count (CBC), urea, creatinine, electrolytes, troponin I, serum lactate dehydrogenase ( LDH), serum protein, and pleural fluid study. If needed, pleural fluid was tested for diagnostic reasons and if needed, a therapeutic drainage was done. From this data, we identified the etiology in CKD patients (on HD or not on HD). We also observed the outcome of patients and conditions of patients on discharge. We divided the PE into exudative or transudative based on the Lights criteria. Exudative PE met at least one of the following criteria, whereas transudative PE met none of the Light's criteria: pleural fluid protein divided by serum protein greater than 0.5, pleural fluid LDH divided by serum LDH greater than 0.6, and pleural fluid LDH greater than two-thirds of the upper normal limit of serum LDH.

The data was entered and analyzed on SPSS version 21 (IBM Corp., Armonk, USA). The cleaning and coding of data were done before analysis. Continuous variables were expressed in mean ± STD, while frequencies with percentages were obtained for categorical variables. The Chi-square test was applied to see the association between variables. A p-value of ≤ 0.05 was considered significant.

## Results

There were 280 patients in the study, of which 158 (56.4%) were male and 122 (43.6%) were female. The mean age was 55.5±14.8 years. Hypertension (HTN) was the most common comorbidity in our patients [241 (86.1%)], while diabetes mellitus (DM) was present in 213 (76%) patients. A previous history of TB was present in 29 (10.4%) patients, and 99 (35.4%) patients had PE in the past. In our study, the majority of patients were not on dialysis (CKD stage II-V) [159 (56.8%)] (Table 1). The signs and symptoms at the time of admission are shown in Table 2.

**Table 1:** Demographic and clinical parameters of 280 patients DM: diabetes mellitus; HTN: hypertension; IHD: ischemic heart disease; TB: tuberculosis; PE: pleural effusion; HD: hemodialysis.

Variables	n (%) / Mean±SD
Male/Female	158 (56.4)/122 (43.6)
Age	55.5 ± 14.8
Positive Smoking history	41 (14.6)
DM	213 (76)
< 5 years	115 (41.1)
≥ 5 years	98 (35)
HTN	241 (86.1)
IHD	76 (27.1)
Liver disease	16 (5.7)
Previous history of TB	29 (10.4)
Treated	15 (5.4)
Untreated	14 (5)
History of malignancy	4 (1.4)
Previous history of PE	99 (35.4)
Unilateral	46 (16.4)
Bilateral	53 (18.9)
Hepatitis B +ve	17 (6.1)
Hepatitis C+ve	14 (5)
CKD stage	159 (56.8)
II-III	20 (7.1)
IV	67 (23.9)
V	72 (25.7)
Hemodialysis	121 (43.2)
one/week	14 (5)
two/week	67 (23.9)
three/week	40 (14.3)
Duartion of HD<1 year	55 (19.6)
Duration of HD≥ 1 year	66 (23.6)

**Table 2:** Signs and Symptoms

Variables	n (%)
Shortness of breath	225 (80.4)
Chest pain	51 (18.2)
Cough	69 (24.6)
Sputum	48 (17.1)
Fever: low grade/ high grade	91 (32.5/11.8)
Weight loss	64 (22.9)
Anorexia	221 (78.9)
Pallor	108 (38.6)
Clubbing	7 (2.5)
Ascites	44 (15.7)
Edema	202 (72.1)

Most of the patients had bilateral PE [234(83.6%)], which was mainly transudative 212 (75.7%) and the most common cause of transudative effusion was fluid overload secondary to CKD [148 (63.2%)]. The most prevalent reason for exudative PE was TB [37(54.4%)]. Diagnostic thoracocentesis was done in 234 (83.6%) patients and the majority of patients [236 (84.3%)] were medically treated. A total of 251 (89.6%) recovered, however, 18 (6.4%) patients died (Table 3). Laboratory parameters of patients are shown in Table 4.

**Table 3:** Detailed clinical status of patients

Clinical variables of patients	n (%) / Mean±SD	
		Bilateral pleural effusion	234 (83.6)	
Unilateral pleural effusion	46 (16.4)	
Transudative effusion	212 (75.7)	
Fluid overload due to chronic kidney disease	148 (63.2)	
Fluid overload due to heart failure	39 (18.4)	
Fluid overload due to nephrotic syndrome	25 (11.8)	
Exudative effusion	68 (24.3)	
Tuberculosis	37 (54.4)	
Uremic pleuritis	21 (30.9)	
Empyema	10 (14.7)	
Diagnostic thoracocentesis done	234 (83.6)	
Therapeutic thoracocentesis done	46 (16.4)	
Normal echocardiogram	169 (60.4)	
Low ejection fraction in echocardiogram	67 (23.9)	
Valvular lesion	44 (15.7)	
Abnormal ECG	35 (12.5)	
Culture done and organism detected	10 (3.6)	
Pseudomonas	5 (50)	
Staph aureius	3 (30)	
Klebsiella	2 (20)	
Total stay in hospital in days	5.8± 2.3	
Medically treated	236 (84.3)	
Intervention required	44 (15.7)	
Improved	255 (91.1)	
Resistant effusion	13 (9.6)	
Recovered	251 (89.6)	
Referred	11 (3.9)	
Death	18 (6.4)	

**Table 4:** Laboratory parameters of patients

Lab parameters	Mean ± Std	Median	IQR	Minimum	Maximum
Hemoglobin	8.4 ± 2	8.9	3.3	5.2	16.8
Total leukocyte count	13 ± 8.9	10.6	9.4	2.9	98
Platelet	252.9 ± 107.3	236	101	16	660
Urea	191.3 ± 64.8	190	75	29	373
Creatinine	7.7 ± 3.3	7	3.4	1.5	23.1
Sodium	133.4 ± 6	133	7	114	156
Potassium	4.7 ± 0.9	4.8	1.4	2.5	8
Chloride	105 ± 5.6	105	10	85	118
Bicarb	17.5 ± 3.6	18	5	6	28
Calcium	8.3 ± 1	8.3	1.1	5.5	11.35
Phosphorous	7.1 ± 2.5	6.8	3.8	2.9	12.9
Albumin	3.2 ± 0.5	3.5	0.5	1.6	5.9

CKD stage was significantly associated with the type of PE. Transudative effusion was predominantly present in patients who had advanced stages of CKD or had End-Stage Renal Disease(ESRD), while exudative effusion was common in patients who were on HD [61 (39.7%)]. A total of 39 (57.4%) patients were on > one year on HD and 30 (44.1%) were having twice-weekly sessions of HD.

Among all comorbidities, only Ischemic Heart Disease (IHD) was significantly associated with the type of effusion. The patients who had IHD primarily suffered from transudative effusion 64 (30.2%) as compared to patients without IHD, who typically developed exudative PE [56 (82.4%)]. Transudative effusion were all bilateral, while exudative was mostly unilateral 46 (67.6%). In both types of effusions, an equal number of patients died, while 201 (94.8%) patients recovered who had transudative effusion and 50 (73.5%) patients improved who had exudative PE (Table 5).

**Table 5:** Association of variables with the type of pleural effusion CKD: chronic kidney disease; DM: diabetes mellitus; HTN: hypertension; IHD: ischemic heart disease

Parameters of patients		Exudate	Transudate	p value
Age	≤ 50 years	18 (26.5)	61 (28.8)	0.289
	51-65 years	24 (35.3)	91 (42.9)	
	> 65 years	26 (38.2)	60 (28.3)	
CKD stage	II- III	1 (1.5)	19 (9)	<0.001
	IV	3 (4.4)	64 (30.2)	
	V	3 (4.4)	69 (32.5)	
	ESRD	61 (89.7)	60 (28.3)	
Hemodialysis duration	Not on HD	7 (10.3)	152 ( 71.7)	<0.001
	< 1 year	22 (32.4)	33 (15.6)	
	≥ 1 year	39 (57.4)	27 (12.7)	
Frequency of Hemodialysis	Not on HD	7 (10.3)	152 ( 71.7)	<0.001
	one/week	8 (11.8)	6 (2.8)	
	two/week	30 (44.1)	37 (17.5)	
	three/week	23 (33.8)	17 (8)	
DM	Yes	53 (77.9)	160 (75.5)	0.678
	No	15 (22.1)	52 (24.5)	
HTN	Yes	60 (88.2)	31 (14.6)	0.554
	No	8 (11.8)	181 (85.4)	
IHD	Yes	12 (17.6)	64 (30.2)	0.043
	No	56 (82.4)	148 (69.8)	
Site of pleural effusion	Unilateral	46 (67.6)	0 (0)	<0.001
	Bilateral	22 (32.4)	212 (100)	
Treatment	Medicine	33 (48.5)	203 (95.8)	<0.001
	Intervention	35 (51.5)	9 (4.2)	
Outcome	Recovered	50 (73.5)	201 (94.8)	<0.001
	Expired	9 (13.2)	9 (4.2)	
	Referred	9 (13.2)	2 (0.9)	

## Discussion

Pleuro-pulmonary problems are very common in the CKD population. On a frequent basis, it is observed that many CKD patients who were admitted due to various causes, had PE either bilateral or unilateral. Keeping in view the increased frequency of PE in CKD patients, we prospectively reviewed the data of these patients and found that in seven months period total of 789 patients were admitted due to various reasons, and out of them, 280 patients had PE. These patients were in different stages of CKD commonly in stage IV [67 (23.9%)] and V [72 (25.7%)] and 121 (43.2%) patients were undergoing HD. Several studies have been done that have evaluated the frequency of PE in the CKD population. One study showed the incidence of PE of 6.7% in various stages of CKD who were not on HD [9], while few studies have reported a higher incidence in patients on maintenance dialysis [6-7].

The mean age of our study subject was 55.48 years with male predominance 56.4% males vs 43.6% females, which was similar to study done by Ray et al in 2013 [9]. In CKD, there are several reasons for PE as these patients are immunocompromised [10-11]. They have decreased cellular and humoral immunity and reduced macrophage and phagocytic activity. Mostly the patients had a history of IHD, which further precipitates the symptoms and these patients mostly have excessive fluids in their bodies. The comorbid conditions associated with or attributed to CKD may indirectly cause pleural abnormalities too. With regard to comorbidities, most patients were diabetic [213 (76%] with 76 patients having a history of IHD. In DM, most patients had proteinuria and these patients developed transudative PE. In a study done among the CKD population having PE, eight out of 31 patients had DM [9] and most of them had a transudative PE, 70% were hypertensive. Similar findings were observed by Prem Kumar et al in which out of 35 patients 62.8% had hypertension followed by DM (42.48%) [12]. In our study, HTN was also the most common comorbid (86.1%).

A previous history of TB was in 10.4% of patients, which is different from a study done in 2015 on 35 CKD patients and concluded that out of them, 11 (31.42%) had a past history of TB [12].

Shortness of breath is the commonest symptom found in these patients. SOB mostly occurs due to the accumulation of fluids in the lungs due to which the lungs are not able to expand properly causing difficulty in breathing. We observed that 225 patients (80.4%) experienced SOB due to PE. Other studies [9-12] have also concluded that SOB is the prime complaint in these patients.

The second common symptom in these patients was cough, which was present in 69 (24.6%) patients and 48 (17.11%) had cough with sputum. A study done in Pune, India showed that cough was the major complaint of their patients [n=22 (79%)] [12]. We found more cases of cough associated with exudative PE, the cough was mild and non-productive.

The third symptom which was faced by these patients was fever (low-grade n=91, 32.5% and high-grade n=33, 11.8%). Fever mostly represents some sort of infection; in our analysis, the patients with TB and empyema experienced more fever as compared to other patients. In one study, 70% of the patients had fever as a presenting complaint [12], while other research showed 15% (n=40)** **[13]. 

Patients with PE usually complain of sharp pleuritic pain during breathing; this occurs because the pleural lining of the lungs is inflamed which causes chest pain. In our study 51 (18.2%) patients had chest pain while contradictory to our analysis, few studies [12-13] have shown 50% of the patients complaining of pleuritic chest pain.

PE may be transudative or exudative depending upon comorbidities and the patient’s condition. Our patients mostly had transudative PE 212 (75.7%) while 68 (24.3%) patients had exudative PE. Many studies had similar findings of a high incidence of transudative PE in CKD patients [13-14]. We found that exudative PE was found in 61 (89.7%) patients who were on HD. One patient was in stage II and III, three were in stage IV, while the remaining three patients were in stage IV who were not on HD; 212 (75.7%) patients who had transudative effusion 69 (32.5%) patients were of stage V (not on HD) and 60 (28.3%) patients were on HD.

A study done in 2007 by Bakirci et al in 2007 showed PE in long term HD patients, they concluded that fluid overload was the commonest cause of PE [7] and the increased volume was due to poor management of fluid balance [15]. Another study done among patients of CKD by Jarrat and Sahn et al [6] shared the observation that hypervolemia was seen in 61.5% of these patients with PE. In our study, among cases of 148 (63.2%) transudative effusions, fluid overload was the commonest, similar to other studies.

After fluid overload, heart failure was the second commonest cause found in our analysis [39 (13.9%)], which is also similar to the Jarrat and Sahn study [6]. Concomitantly, a study done by Bakirci et al [7] found the incidence of heart failure to be 9.6% in their patients. Another study done in 430 CKD patients in 2013 found 31 had PE (29 patients were of different stages of CKD and 2 post-renal transplant patients) and among them, 15 patients had transudative PE of which 13 patients had heart failure [9].

Current theories postulate that mostly patients with Congestive Heart Failure (CHF) have left ventricular failure, which consequently leads to PE. The high amount of fluid in the interstitial spaces [16] enters the pleural spaces through the highly permeable visceral pleura [17] and consequently causes PE.

Echocardiographic findings in our study showed that 67 (23.9%) patients had low Ejection Fraction (EF) and left ventricular hypertrophy, and among them, 24 (35.8%) patients had PE due to heart failure, which was transudative in origin.

In our study, PE due to CHF produced SOB, pedal edema, orthopnea and paroxysmal nocturnal dyspnea, and chest X-ray which mostly showed bilateral PE. A study done by Kumar et al in 2015 [12] among 35 CKD patients concluded that 31.42% of patients developed PE due to heart failure.

We found that transudative effusions due to any cause were bilateral [212 (75.7%)] and exudative effusions were unilateral [46 (16.4)], which is also similar to a study by Bakirci et al [7], which also showed bilateral PE with transudative etiology (66.7%).

A study done by Kumar et al [12] among 35 patients showed bilateral transudative effusions in 11 (31.42%) patients and exudative effusions were 24 (68.57%) out of which seven (29.16%) were bilateral and 17 (20.83%) were unilateral.

In our study we found three major causes for exudative effusion: TB was the most frequent cause 37 (80.4%) The association of TB with immunocompromised states like ESRD patients is well known for many years [18-19]. Many studies support the increased rate of TB in CKD patients, which causes exudative PE. While opposite to our study, Bakirci et al [7] showed only 1.9% of TB. Another study by Kumar et al showed 28.5% of cases of PE due to TB [12].

The characteristic feature of exudative effusion is serosanguinous fluid with increased lymphocytes [20] and its frequency has been reported in 1 to 58% of the patients with ESRD [21-22]. It is difficult to confirm TB in these patients because defective cell-mediated immunity caused difficulty in detecting mycobacterium TB and in many patients empirically antituberculous therapy started to cure the symptoms.

A worldwide summary article demonstrated a 6.9 to 52.5 fold increased risk of TB in CKD patients [20]. In India, the incidence of TB in HD patients has been documented at around 8.7% [12].

The second cause of exudative effusion in our study was uremic pleurisy [21 (30.9%)] and 19 patients were of HD. Other causes were empyema eight (2.9%) and two (0.7%) patients had parapneumonic effusion. Uremic pleurisy was first reported in 1955 by Hopps and Wissler in 20% of uremic patients at autopsy [23]. It is a diagnosis of exclusion. In our study, 21 patients had uremic pleurisy - the diagnosis was made on the basis of exudative effusion with lymphocytic predominance mostly [7 (10.4%)]. Among these patients, most were on twice-weekly HD, eight patients were on thrice-weekly, but their duration of dialysis of each session was 2.5 to 3 hours, which is less than adequate.

The pathogenesis of uremic pleurisy is still not clear, but toxins like uremic acid, phosphates and retained immune complexes might be implicated in its pathogenesis [24]. Uremic effusions can occur at any time and it has no specific association with the degree of uremia [5]. Uremic effusion can recur or progress despite improvement in dialysis frequency and duration. A study done in 2013 [9] showed 19.4% (six out of 31 cases) cases of uremic pleural effusion in CKD patients.

The third cause of exudative effusion was empyema, which is pus in the pleural space. We also found some cases of parapneumonic effusion, which is any effusion secondary to pneumonia, and can be unilateral or bilateral. A study done by Bakirci et al on 52 patients showed that 9.6% had parapneumonic effusion [7].

As already explained that total 10 patients developed empyema and parapneumonic effusion with different organisms growing in their pleural fluid culture. They needed drainage with chest tube placement. Among 10 patients, seven patients were on HD, two were in CKD stage IV and one in CKD stage IV who was not on dialysis. Due to reduced overall immunity, parapneumonic effusion and empyema are common in the CKD population [22].

Anaerobic gram-negative organisms [25] and aerobic gram-positive organisms [22] are the predominant pathogens in CKD patients. Organisms that grew in pleural fluid culture in our study included five cases of pseudomonas (50%), three cases of *Staphylococcus aureus *(30%) and two of *Kleibseilla pnemonii *(20%). This observation is slightly consistent with the study done by Ray et al [9], which also concluded similar results in their HD patients.

## Conclusions

PE is common in CKD patients mainly in stages IV and V. These patients are immunocompromised due to reduced cell-mediated immunity. There are several etiologies for PE in CKD patients. Transudative and exudative both are common in these patients. Early diagnosis is mandatory to cure the cause. SOB is the most common clinical presentation; fluid overload and heart failure were the commonest causes of bilateral transudative PE; and TB was the common cause of exudative PE. In hemodialysis patients, PE most commonly occurs due to underdialysis state, which needs proper thrice weekly dialysis for longer duration.
